# Numerical response of mammalian carnivores to rodents affects bird reproduction in temperate forests: A case of apparent competition?

**DOI:** 10.1002/ece3.4608

**Published:** 2018-11-20

**Authors:** Alex Grendelmeier, Raphaël Arlettaz, Gilberto Pasinelli

**Affiliations:** ^1^ Swiss Ornithological Institute Sempach Switzerland; ^2^ Division of Conservation Biology, Institute of Ecology and Evolution University of Bern Bern Switzerland; ^3^ Swiss Ornithological Institute, Valais Field Station Sion Switzerland; ^4^ Department of Evolutionary Biology and Environmental Studies University of Zurich Zurich Switzerland

**Keywords:** apparent competition, incidental prey, mast seeding, numerical response, predation

## Abstract

Resource pulses such as mast seeding in temperate forests may affect interspecific interactions over multiple trophic levels and link different seed and nonseed consumers directly via predation or indirectly via shared predators. However, the nature and strength of interactions among species remain unknown for most resource pulse–driven ecosystems. We considered five hypotheses concerning the influence of resource pulses on the interactions between rodents, predators, and bird reproduction with data from northern Switzerland collected between 2010 and 2015. In high‐rodent‐abundance‐years (HRAYs), wood warbler (*Phylloscopus sibilatrix*) nest survival was lower than in low‐rodent‐abundance‐years, but rodents were not important nest predators, in contrast to rodent‐hunting predators. The higher proportion of nests predated by rodent‐hunting predators and their increased occurrence in HRAYs suggests a rodent‐mediated aggregative numerical response of rodent‐hunting predators, which incidentally prey on the wood warbler's ground nests. There was no evidence that rodent‐hunting predators responded behaviorally by switching prey. Lastly, nest losses caused by nonrodent‐hunting predators were not related to rodent abundance. We show that wood warblers and rodents are linked via shared predators in a manner consistent with apparent competition, where an increase of one species coincides with the decrease of another species mediated by shared predators. Mast seeding frequency and annual seed production appear to have increased over the past century, which may result in more frequent HRAYs and generally higher peaking rodent populations. The associated increase in the magnitude of apparent competition may thus at least to some extent explain the wood warbler's decline in much of Western Europe.

## 1 INTRODUCTION

Many ecosystems worldwide are driven by intermittent resource pulses, a sudden increase in resources often synchronized temporally and spatially over large geographic areas (Yang et al., 2010). Due to their influence at multiple trophic levels, resource pulses can cause strong growth and decline of populations of various taxa (Jaksić, Silva, Meserve, & Gutiérrez, 1997; Jȩdrzejewska & Jȩdrzejewski, 1998; King, 1983; Schmidt & Ostfeld, 2008). In temperate forest ecosystems, mast seeding of various tree species constitutes a primary intermittent resource pulse (Lalonde & Roitberg, 1992), which permeates throughout the food web by triggering various subsequent pulses (Clotfelter et al., 2007; Yang et al., 2010). A prominent secondary pulse following mast seeding consists of a demographically based numerical response (increase in numbers via reproduction) by seed consuming small rodents. Rodents respond to mast seeding with increased overwinter survival and overwinter breeding (in addition to spring and summer breeding), and thus have larger spring and summer populations than in years without preceding mast seeding (Jensen, 1982; Ostfeld, Jones, & Wolff, 1996; Pucek, Jȩdrzejewski, Jȩdrzejewska, & Pucek, 1993). Annually varying rodent numbers may affect occurrence and distribution of rodent‐hunting predators, which may exhibit a rodent‐mediated numerical response (Francksen, Whittingham, Ludwig, Roos, & Baines, 2017; McKinnon, Berteaux, Gauthier, & Bêty, 2013; McShea, 2000; Mills, 2012; Schmidt & Ostfeld, 2003). In Scottish moorlands for instance, buzzards (*Buteo buteo*) responded numerically to high rodent abundances and thereby incidental predation by buzzards on red grouse (*Lagopus lagopus*) increased as well (Francksen et al., 2017). Alternatively, predators may not respond numerically to varying rodent numbers, but behaviorally, by switching prey when rodents become scarce (prey switching causes type 3 functional response, e.g., Abrams & Matsuda, 2003; Jȩdrzejewska & Jȩdrzejewski, 1998; Murdoch, 1977). In Białowieża National Park, Poland, carnivores switched from hunting rodents as their main prey to alternative prey such as blackbirds (*Turdus merula*) and their nests, causing low breeding success in years of rodent scarcity (Jȩdrzejewska & Jȩdrzejewski, 1998). Lastly, the type of response seems to depend on the predator species and/or time since the primary resource pulse (Schmidt & Ostfeld, 2003).

Because of shared predators, various other species may experience increased predation pressure as a consequence of past mast seeding, such as amphibians, reptiles, insectivores, insects, and birds (Brangi, 1995; Clotfelter et al., 2007; Jȩdrzejewski & Jȩdrzejewska, 1993; Jȩdrzejewski, Jȩdrzejewska, Zub, Ruprecht, & Bystrowski, 1994; Martin, 1995; Schmidt & Ostfeld, 2003; Sidorovich, Sidorovich, & Krasko, 2010). Resource pulses can hence not only directly influence fitness and population dynamics of seed consumers (primary prey in the case of rodents) and predators of seed consumers, but also of species considered to be alternative (secondary prey actively searched for; Holt, 1977) or incidental prey (secondary prey not actively searched for; Cornell, 1976).

Resource pulse dynamics and their effects at various trophic levels have received some attention (Yang et al., 2010). Studies examining the relative importance, role, and response type of different species involved in predator–prey systems in resource pulse–driven ecosystems are scarce. Furthermore, understanding how population dynamics of seed and nonseed consumers respond to changing mast seeding dynamics is important, as there is evidence for increases in mast seeding frequency (Övergaard, Gemmel, & Karlsson, 2007; Paar, Guckland, Dammann, Albrecht, & Eichhorn, 2011) and overall larger seed crops (Callahan, Del Fierro, Patterson, & Zafar, 2008; Gatter, 2000; Hilton & Packham, 2003) over the last decades.

**Figure 1:**
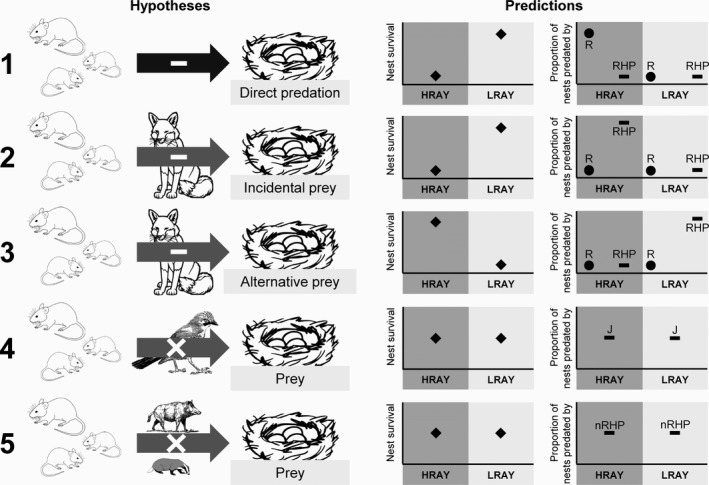
Hypotheses and predictions. Shown are the five hypotheses considered, with predictions for nest survival (squares) in “high‐rodent‐abundance‐years” (HRAY) and “low‐rodent‐abundance‐years” (LRAY), as well as for the proportion of nests predated by different predator groups during HRAY and LRAY. “R” = rodents (dots), “RHP” = rodent‐hunting predators (bar), “J” = jays (bar), “nRHP” = nonrodent‐hunting predators (bar). Black arrow = direct negative effect. Gray arrow over predator group = indirect effect via this predator group. Negative sign in arrow = negative effect expected. X in arrow = no effect expected

In Europe's temperate forests, rodents, rodent‐hunting predators, but also nonrodent‐hunting predators alongside with various alternative and incidental prey species are part of resource pulse systems based on mast seeding of deciduous tree species such as European beech (*Fagus sylvatica*), oak (*Quercus* spp.), or hornbeam (*Carpinus betulus*). It is well established that settlement and occurrence of the ground nesting wood warbler (*Phylloscopus sibilatrix*), a small songbird occurring in such deciduous forests, are negatively correlated with rodent abundance (Pasinelli, Grendelmeier, Gerber, & Arlettaz, 2016; Szymkowiak & Kuczyński, 2015; Wesołowski, Rowiński, & Maziarz, 2009), though the exact mechanism underlying the avoidance of rodent‐rich habitat by this passerine is unknown. Rodents may be perceived as direct threat or taken as proxy for general predation risk arising from rodent‐hunting predators. We considered five competing hypotheses (Figure 1) which may explain the relationship between wood warbler nest survival and rodent abundance in mast‐driven forest ecosystems: (1) rodents influence wood warbler nest survival directly via predation (rodents are the main predators), (2) rodents influence wood warbler nest survival indirectly by triggering a numerical (aggregative; Mills, 2012) response of rodent‐hunting predators, which incidentally depredate wood warbler nests (incidental prey), or (3) rodents influence wood warbler nest survival indirectly by triggering a behavioral response in rodent‐hunting predators, which switch from rodents to wood warbler nests (alternative prey; type 3 functional response). In addition, we also assessed the role of nest predators not associated with rodents. An important nest predator in Western Europe is the Eurasian Jay (*Garrulus glandarius*, jay hereafter) (Grendelmeier, Arlettaz, Gerber, & Pasinelli, 2015; Mallord et al., 2012). However, because jays are not linked to rodents, we hypothesized that (4) rodents do not influence wood warbler nest survival via jay predation. Likewise for the remaining nest predators not linked to rodents, but being of much lower importance as nest predators than jays, we hypothesized that (5) rodents do not influence wood warbler nest survival via predation from the remaining nonrodent‐hunting nest predators. In other words, hypotheses 4 and 5 state that predation on wood warbler nests by neither jays nor the remaining nonrodent‐hunting predators is mediated by rodents.

## 2 METHODS

### 2.1 Study area and species

The study ran from 2010 and 2015 and took place in 14 areas along northern Switzerland's Jura mountain chain as well as in one area near Lake Constance and one area in the prealpine valley of Glarus (median distance between study areas = 24 km; Table S1). Generally, the study areas were located on slopes exposed to the south and consisted of mixed deciduous forest stands dominated by European beech, with other deciduous and coniferous tree species interspersed. Stands predominantly consisted of old polewood and young timber with a relatively closed canopy and a sparse shrub layer, if at all present.

The wood warbler has suffered long‐term declines in many EU countries since at least 1980 (Vickery et al., 2014). In Switzerland, it has been red‐listed as vulnerable (Keller, Gerber, Schmid, Volet, & Zbinden, 2010) and is considered a priority species for the Swiss species recovery program for breeding birds (Keller, Ayé, Müller, Spaar, & Zbinden, 2010). This insectivorous forest‐interior passerine winters south of the Sahara desert (Hobson et al., 2014) and exhibits very little natal and breeding site fidelity (Wesołowski et al., 2009). Strong annual fluctuations of local population sizes have been shown, with decreased settlement and occurrence in areas with high rodent populations (Pasinelli et al., 2016; Szymkowiak & Kuczyński, 2015; Wesołowski et al., 2009).

### 2.2 Assessing wood warbler reproduction

From April to July, each study area was visited twice a week to map singing males, pairs, and nests. Whenever possible nests were monitored with one trail camera (Reconyx PC900 HyperFire Professional High Output Covert; Reconyx, Inc., Holmen, WI, USA), allowing to survey activity of adults and (old) nestlings, to identify nest predators and to determine the exact date of nest predation or fledging and hence exposure time (see Section 2.7). Detailed descriptions on how nest status, first egg date, hatching date, nestling age, and fledging date were determined, are outlined in Grendelmeier et al. (2015). Once a nest was inactive (successful or unsuccessful), we measured rodent abundance and several environmental factors (details given below) around the nest.

### 2.3 Assessing rodent abundance

**Figure 2:**
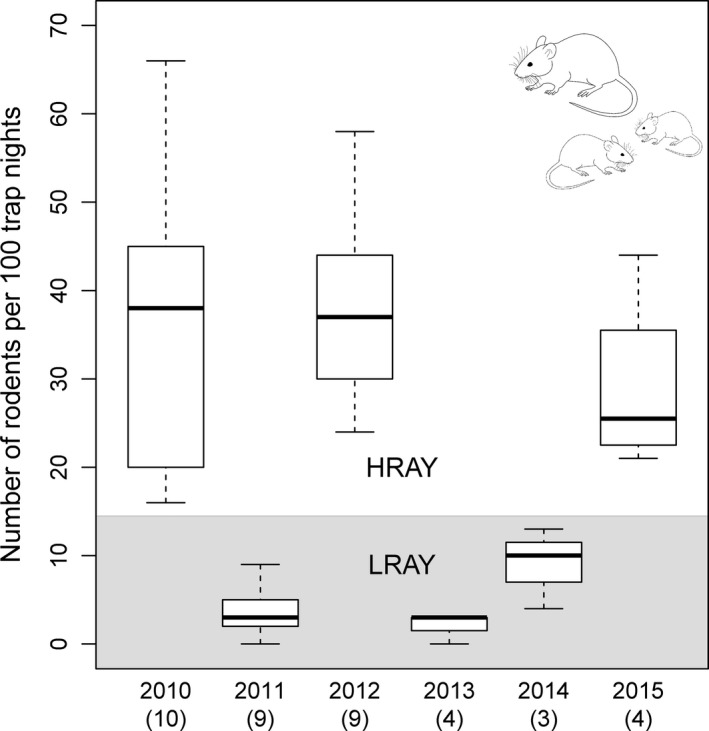
Variation in rodent abundance between years. Depicted is the number of rodents per 100 trap nights for each of the six study years, with medians (bold line), quartiles (box) and ranges (whiskers), as well as number of study areas in parentheses. We categorized rodent abundances based on ranges (whiskers) into HRAY (white area) and LRAY (gray area)

We used live‐trapping of rodents in breeding territories (968 m^2^) of wood warblers and in control areas (of equal size) without wood warblers and with the center located 200 m away from the breeding territory center (i.e., the nest) in a random direction but within the same study forest patch (paired design and called "plot pair" hereafter; Pasinelli et al., 2016). A paired design was used instead of study area‐wide trapping grids to also accommodate other studies within the same project that required a territory‐based approach. Even though 245 territories with nests were available, due to logistical reasons, rodents could only be captured in a total of 125 plot pairs based on the same standardized sampling design (Pasinelli et al., 2016). We used “Longworth traps” (Penlon Ltd., Abingdon, UK) and “Field Trip Trap Live Catch Trap” (Alana Ecology, Bishops Castle, UK), which have similar trapping efficacy (Arlettaz, Krähenbühl, Almasi, Roulin, & Schaub, 2010). Following Pasinelli et al. (2016), we conducted one trapping session with 25 traps each in both the breeding territories and the control areas. Trapping usually started mid‐June and was done during 8 weeks in 2010, during 6 weeks in 2011–2012 and during 5 weeks in 2013–2015. A trapping session lasted 48 hr, during which a closed population was assumed, with 12‐hr intervals between trap checks. Caught animals received interval‐specific markings that allowed distinguishing recaptures from newly caught individuals per trapping session. Due to very low numbers of rodents, densities and capture probabilities could not be calculated for three of 6 years (i.e., in the three low‐rodent‐abundance‐years, hereafter LRAY), and we thus used the number of individuals caught once as measurement of rodent abundance for every year. We found a strong correlation (Spearman rank correlation, *r*_s_ = 0.94, *p* < 0.001, *n* = 54 capture plots) between the number of caught individuals (i.e., omitting recaptures) and density (based on capture‐mark‐recapture analyses in program CAPTURE [v6.0]; Table S2) for captures in 2010, a high‐rodent‐abundance‐year (HRAY hereafter). We are therefore confident that the number of caught individuals is an adequate proxy for rodent abundance. Due to the large disparity between the number of found nests (*n* = 245) and the number of plot pairs where rodents could be captured (*n* = 125), we aggregated all rodent data to categorize each year into a HRAY or a LRAY. It can be assumed that rodent outbreaks and crashes are spatially synchronized over our study area. This assumption is based on (a) the strong correlation (*r*_s_ = 0.92, *p* = 0.008, *n* = 6 years) between rodent numbers and beech mast in the previous year (beech mast data from Burkart, 2016), and (b) the generally strong spatial synchrony of a tree species’ mast seeding over a large geographic area (Ascoli et al., 2017; Koenig & Knops, 1998; Nussbaumer et al., 2016; Pucek et al., 1993). Hence, rodent numbers in our study sites during the same year were either high in all wood warbler territories or low in all wood warbler territories. Therefore, by aggregating and classifying all rodent data into HRAY and LRAY, the loss of information concerning rodents seems minimal and was far outweighed by the gain of information from using data from all 245 nests compared to only 125. To achieve the classification of HRAY and LRAY, we summed the number of caught individuals per study area and year, divided the sum by the corresponding total number of trap nights, and multiplied by 100 to obtain number of rodents per 100 trap nights per study area and year. Based on annual ranges (whiskers in Figure 2) of number of rodents per 100 trap nights, we then classified our six study years into LRAY and HRAY, respectively, and called the variable categorical rodent abundance (CRA hereafter) for all subsequent analyses. The rodent species caught in our study were *Apodemus* mice (not identified to species level, but most likely *A. flavicollis* and *A. sylvaticus*, 58.2%) and bank voles (*Myodes glareolus*, 39.6%), with very rare captures of *Glis glis* (1.3%), *Microtus agrestis* (0.7%) and *Mus musculus* (0.2%).

### 2.4 Assessing proportion of nests predated by different predator groups

In this study, we looked at four predator groups. (a) Rodents consisted of the six “small rodent” species described in the previous paragraph. (b) Rodent‐hunting predators (RHP hereafter) comprised the pine marten (*Martes martes*), the red fox (*Vulpes vulpes*), the stone marten (*Martes foina*), and the tawny owl (*Strix aluco*). We did not count house cats (*Felis catus*) as RHP, because we assume that their distribution and hunting behavior are strongly influenced by humans, and because they are not important nest predators in Switzerland (this study; Grendelmeier et al., 2015), in Germany (Stelbrink, 2016), in Wales and England (Bellamy et al., 2018; Mallord et al., 2012; Maziarz, Piggott, & Burgess, 2017) or in Poland (Maziarz et al., 2018). During 4 years, only four Eurasian lynx (*Lynx lynx*) and two European wildcats (*Felis silvestris silvestris*) could be photographed, but neither species depredated nests. (c) Jays, despite not being linked to rodents, are important predators of wood warbler nests in Western Europe (Mallord et al., 2012; Grendelmeier et al., 2015; this study) and were therefore assessed as separate predator group. (d) nonrodent‐hunting predators (nRHP hereafter) consisted of the European badger (*Meles meles*), the Eurasian sparrowhawk (*Accipiter nisus*), the common blackbird, the house cat, the honey buzzard (*Pernis apivorus*), the wild boar (*Sus scrofa*), and the Eurasian red squirrel (*Sciurus vulgaris*).

The proportion of nests predated by the different predator groups was expressed as the number of predation events by a predator group divided by the total number of predation events per study area and year. Calculations were based on a sample of nests where predators could be identified (*n* = 78). Twenty‐one of 99 predated nests had to be omitted from the analysis due to the unknown predator identity. In 12 of 99 predation events, predators could not be identified because no camera could be installed (high risk of theft). In nine of 99 predation events, predators could not be identified because the camera did not trigger (not because of malfunction, but due to the specific triggering mechanism). Despite not knowing the predator identity, these 21 nests could be classified as predated because the nests were empty before the earliest possible fledging date.

### 2.5 Assessing abundance of rodent‐hunting mammals

Abundance of rodent‐hunting mammals (rodent‐hunting predators excluding tawny owls due to a lack of data, RHM hereafter) was assessed at the level of the study area to examine the possible links between rodent and predator abundance. Abundance of RHM was estimated based on four camera traps installed along one line‐transect in each study area between April and July of the years 2012–2015. Transects spanned the core of the study area (determined based on wood warbler nest coordinates of the years 2010–2011), with cameras spaced roughly 200 m apart, always mounted on the same trees and facing the same direction. This sample design gives a relative abundance index (“abundance” for simplicity), which is adequate for comparative purposes as used in this study. Following O'Brien, Kinnaird, and Wibisono (2003), we assumed pictures of the same species on the same transect (meaning all four cameras per study site) to be independent when at least 30 min between detections on consecutive cameras had elapsed. For each study area and year, we summed all sightings by cameras of RHM and divided them by the summed number of days all transect cameras in a study area and year were recording (mean number of days ± *SD* over all areas and years: 292.3 ± 55.8). The result was multiplied by 100 to obtain number of RHM during 100 camera nights per study area and year. Identification of martens to species level was not possible for transect‐camera pictures, which, compared to cameras monitoring nests (species identification possible), monitored at greater distances, where details for identification to species level can often not be seen anymore.

### 2.6 Assessing grass tussocks and nest concealment

The number of grass tussocks close to the nest and nest concealment has been found to be important environmental variables in relation to wood warbler nest survival (Grendelmeier et al., 2015). To account for their potential effect on nest survival here as well, we included both covariates in our analyses (see below). The covariate “number of grass tussocks” was based on counts of grass tussocks within the same standardized sampling design used for rodent captures, described in detail by Pasinelli et al. (2016). “Nest concealment” was a discrete covariate ranging from 0 to 5 denoting nest concealment from five viewpoints (0 = visible from five sides, hence completely visible; 5 = not visible from any of the five sides, hence completely concealed). For more details on nest concealment, refer to Grendelmeier et al. (2015).

### 2.7 Statistical procedures

#### 2.7.1 Relationship between nest survival and rodent abundance

To assess our predictions concerning the relationship between wood warbler nest survival and rodent abundance (Figure 1), we performed survival analysis using the Cox mixed effects model function implemented in R (R Development Core Team, 2013) with the package “coxme”(Terhneau, 2018). The Cox models is a semiparametric regression model which simultaneously evaluates the effects of several factors on survival and is expressed by the hazard function denoted by *h*(*t*). *h*(*t*) is estimated by *h*_0_(*t*) × exp(*b*_1_ *x*_1_ + *b*_2_ *x*_2_ + … + *b*_p_ *x*_p_) where the hazard *h*(*t*) can vary over time and *h*_0_ is the baseline hazard, which is the hazard value if all covariates equal zero. The impact of the covariates (*x*_1_, *x*_2_, …,*x*_p_) can be measured by their effect size exp(*b*_1_, *b*_2_, …, *b*_p_) (Cox, 1972). As with all time‐to‐event methods, Cox models are designed to correctly incorporate censored data, which in our case arose from nests not found before the first egg was laid (left‐censored data) and predated nests without cameras (interval‐censored data). Exposure time (Julian days between nest finding and nest success/failure) and the end status (successful or failed) of each nest (*n* = 245 nests, “large dataset”) made up the response variable (nest survival), which was modeled in relation to CRA as the only fixed effect (reduced model). Success/failure dates were either based on true dates (nests with cameras) or the median date between the last visit with eggs or nestlings and the final visit without eggs or nestlings inside the nest. We included a random effect for study site to account for the data dependency arising from using the same study sites in multiple years.

**Figure 3:**
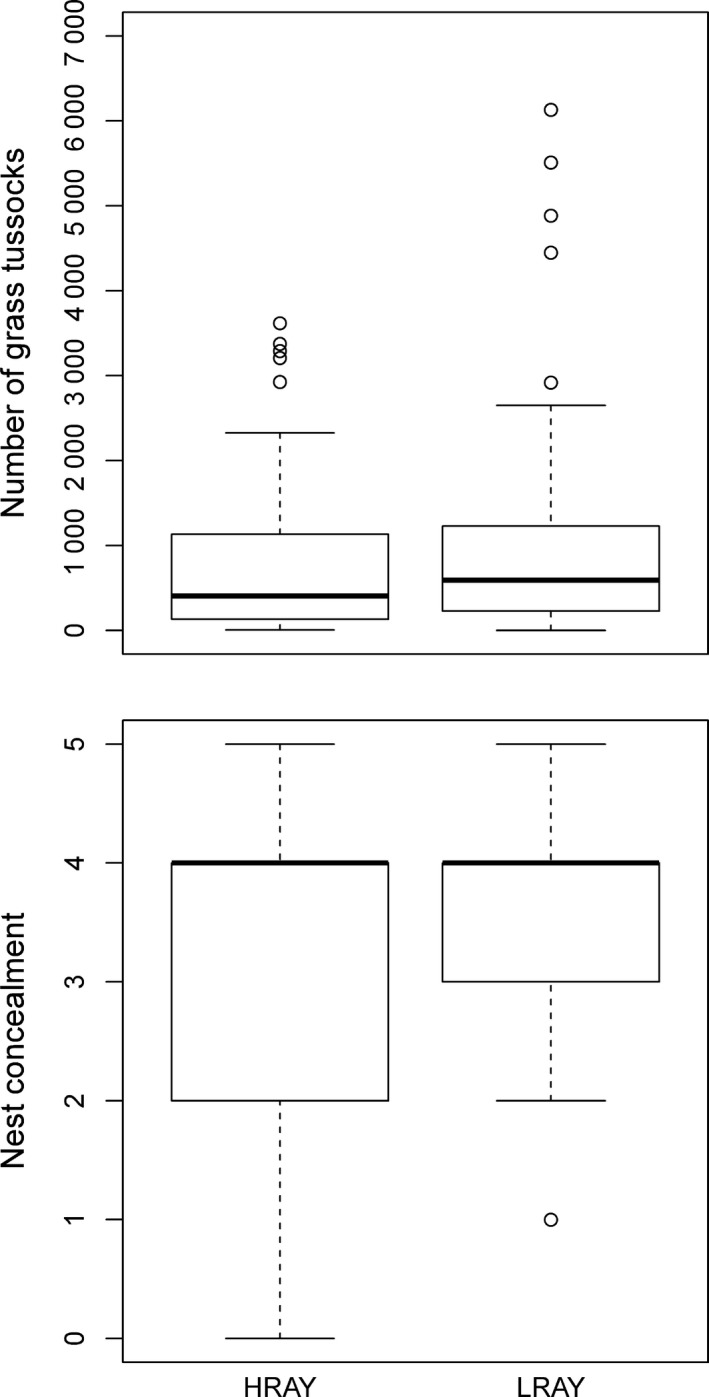
Grass tussocks and nest concealment in relation to rodent abundance. Box plots show “number of grass tussocks” (top panel) and “nest concealment” (bottom panel) in relation to high‐rodent‐abundance‐years (HRAY) and low‐rodent‐abundance‐years (LRAY)

We also assessed nest survival in relation to CRA together with “number of grass tussocks” and “nest concealment” (extended model, Figure 3). Because “number of grass tussocks” was only available for 110 nests (“small dataset” with data from all 16 study areas used in the “large dataset”), we had to fit a separate survival model with these variables. We modeled nest survival in relation to CRA, “number of grass tussocks” and “nest concealment” as fixed effect and again included study area as random effect.

#### 2.7.2 Relationship between the proportion of nests predated by different predator groups and rodent abundance

To investigate the importance of predation by the four predator groups on reproductive success in relation to rodent abundance (Figure 1), we assessed whether the proportion of nests predated by the four predator groups varied between HRAY and LRAY. We modeled the proportion of nests predated by predator groups per study area and year in relation to CRA as fixed effect and study area as random effect, using GLMM with a logit link and binomial error (package lme4; Bates, Maechler, Bolker, & Walker, 2015). Model fit was visually assessed with residual plots. We used Bayesian 95% credible intervals (CrI) to estimate uncertainty of model parameters *β* (Korner‐Nievergelt et al., 2015). Based on the posterior distribution obtained through the function “sim” (R package "arm," Gelman & Su, 2016), we calculated estimates of model parameters from means of the simulated values and 95% CrI from 2.5% and 97.5% quantiles. We also calculated the posterior probability that the difference in the values of the response variable for HRAY and LRAY was different from zero. A parameter *β* was considered to be significant if its 95% CrI did not include 0, thus if the posterior probability was larger than 0.975 (Korner‐Nievergelt et al., 2015).

#### 2.7.3 Relationship between abundance of RHM and rodent abundance

Based on results from the above two analyses, we ran a post hoc analysis to further assess whether RHM directly correlated with rodent abundance. We modeled abundance of RHM with a linear mixed effects model in relation to CRA as fixed effect and study area as random effect. In addition, we ran the same analysis separately for the response variables abundance of red foxes and abundance of martens (abundance of RHM split into the two taxa red foxes and martens). Model fit was visually assessed with residual plots. We again used Bayesian 95% CrI to estimate uncertainty of model parameters *β* and calculated posterior probabilities (as described above). All statistical analyses were conducted in R version 3.4.3 (R Development Core Team, 2013).

## 3 RESULTS

### 3.1 General

During 6 years (2010–2015), we found 245 wood warbler nests, of which 99 were predated (nests empty before possible fledging date), 22 failed due to unknown cause (not nest predation as eggs or nestlings were found dead in nest), and three had unknown fate. As nest predators, we recorded 23 pine martens (30% of identified nest predation events), 18 Eurasian jays (24%), 13 red foxes (17%), seven European badgers (10%), three tawny owls (4%), three Eurasian sparrowhawks (4%), two stone martens (3%), two *Apodemus* mice (3%), one common blackbird (1%), one house cat (1%), one honey buzzard (1%), one wild boar (1%), and one Eurasian red squirrel (1%). Small rodents accounted for only 3% of all confirmed predation events over the 6 years and were thus not important nest predators, neither in the three HRAY nor in the three LRAY. RHP, especially red fox and pine marten, on the other hand were the principal nest predators accounting for 54% of all recorded predation events in our study.

### 3.2 Relationship between nest survival and rodent abundance

**Figure 4:**
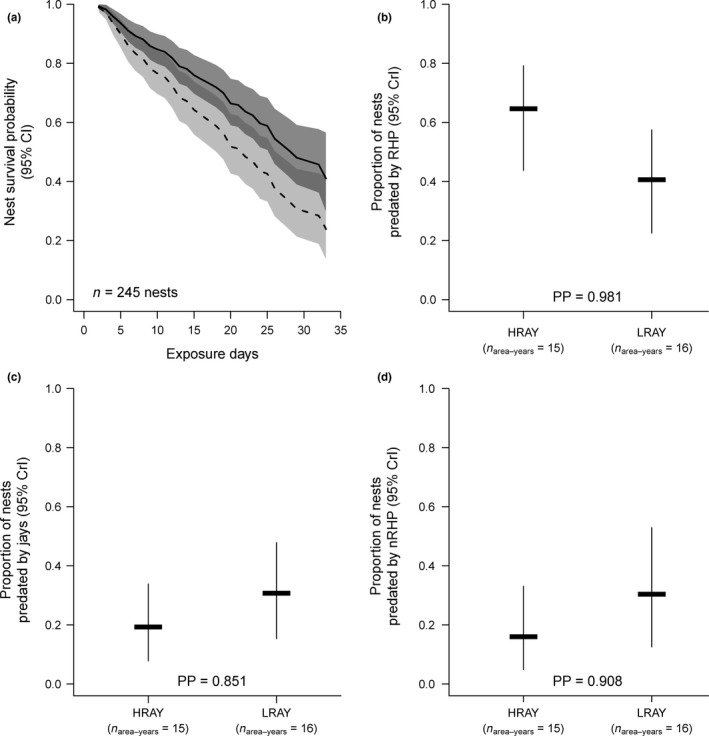
Wood warbler nest survival and proportion of nests predated by rodent‐hunting predators in relation to rodent abundance. Panel (a) shows nest survival probability (lines) and 95% confidence intervals (CI, shaded areas) as a function of HRAY (dashed line and light gray‐shaded area, respectively) and LRAY (solid line and dark gray‐shaded area, respectively) based on the reduced Cox proportional hazard model. Panels (b–d) show the mean fitted values with 95% CrI from the GLMM for the proportion of nests predated by rodent‐hunting predators (RHP), jays and the remaining nonrodent‐hunting predators, respectively, in relation to HRAY versus LRAY. Also shown in panels (b–d) are the respective posterior probabilities (PP, from 0.5 to 1) that the corresponding differences between HRAY and LRAY are different from zero. A parameter *β* was considered to be significant if its 95% CrI did not include 0, thus if the PP was larger than 0.975

For the reduced model, the regression coefficient of LRAY in relation to HRAY (base line) was −0.48. The hazard ratio (exponentiated regression coefficient also representing the effect size) of 0.62 at a *p*‐value of 0.013 indicates a significant relationship between nest survival and CRA, with a hazard reduced by 38% when nesting in LRAY. Hence, wood warbler nest survival was higher in LRAY compared with HRAY and generally decreased over the nesting phase (*n* = 245 nests, Figure 4a).

In the extended model (*n* = 110 nests), the hazard ratios and *p*‐values for rodent abundance were 0.67 (*p* = 0.17), for “number of grass tussocks” 0.78 (*p* = 0.19) and for “nest concealment” 0.82 (*p* = 0.14). Hence, all three covariates contributed only little to the difference in the hazard ratios. Final conclusions concerning nest survival were drawn from the reduced model for three reasons. (a) Results from the reduced model were based on a dataset almost twice as large as the dataset used for the extended model. The use of the much larger dataset thus resulted in considerably increased statistical power to detect differences in nest survival between HRAY and LRAY. (b) The effect size of CRA in the reduced model was similar to the effect size in the extended model (0.62 compared to 0.67), meaning that a similar amount of variance was explained by CRA in either model. (c) There was no evidence for collinearity between “nest concealment” and “number of grass tussocks” (Spearman rank correlation, *r*_s_ = 0.01, *p* = 0.9, *n* = 110 territories). There was also no difference of “number of grass tussocks” (Wilcoxon rank sum test, *W* = 1,362, *p* = 0.38, *n* = 110) or “nest concealment” (*W* = 1,426, *p* = 0.59, *n* = 110) between HRAY and LRAY (Figure 3).

### 3.3 Relationship between the proportion of nests predated by the different predator groups and rodent abundance

As described above, nest predation by rodents was very uncommon, and hence, the proportion of nests predated by rodents in HRAY and LRAY could not be analyzed.

The proportion of nests predated by RHP was higher in HRAY (mean: 0.63, CrI: 0.43–0.79) compared with LRAY (mean: 0.39, CrI: 0.22–0.58), with support for a significant difference between HRAY and LRAY (posterior probability of 0.981 being above the significance threshold [see Section 2.7.2]; Figure 4b). There was no significant difference in the proportion of nests predated by jays (posterior probability of 0.851 being below the significance threshold, Figure 4c) or the proportion of nests predated by nRHP (posterior probability of 0.908 being below the significance threshold, Figure 4d) between HRAY (jays: mean = 0.17, CrI = 0.08–0.36; nRHP: mean = 0.14, CrI = 0.04–0.35) and LRAY (jays: mean = 0.29, CrI = 0.15–0.48; nRHP: mean = 0.28, CrI = 0.12–0.54).

### 3.4 Relationship between abundance of RHM and rodent abundance

**Figure 5:**
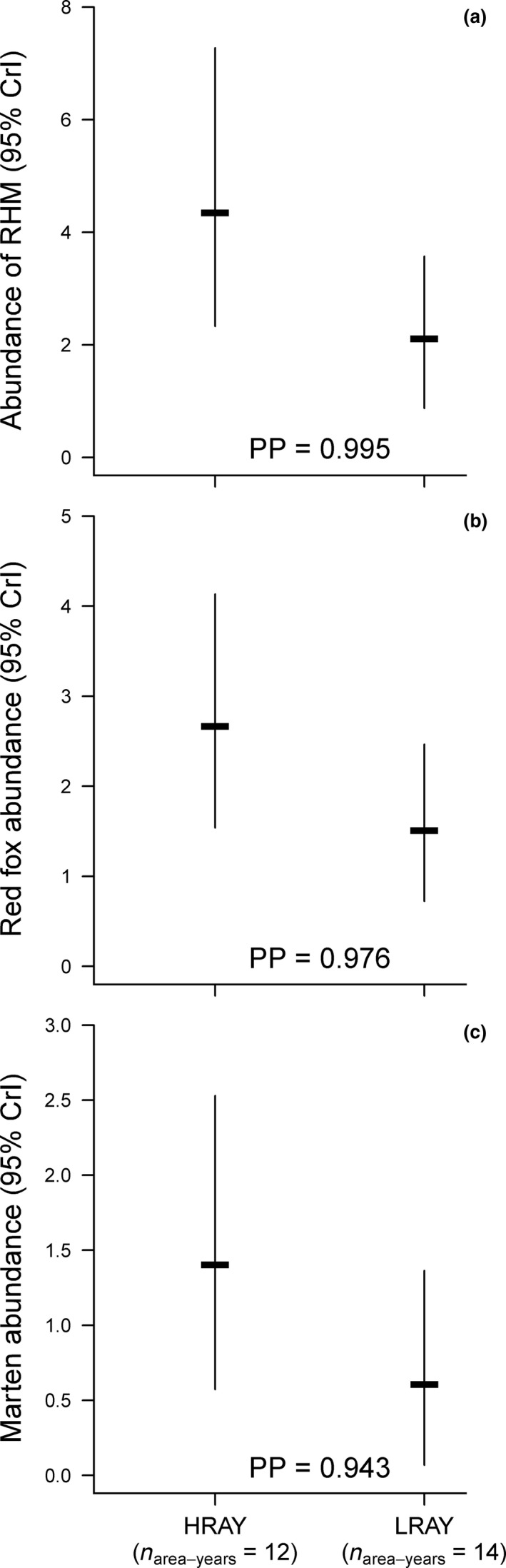
Relationship between predator abundance and rodent abundance. Panels show mean fitted values and 95% CrI of the abundance of (a) RHM, (b) red fox and (c) marten in relation to HRAY versus LRAY. Also, given is the corresponding posterior probability (PP, from 0.5 to 1) that the difference between HRAY and LRAY is different from zero. A parameter *β* was considered to be significant if its 95% CrI did not include 0, thus if the PP was larger than 0.975

The abundance of RHM was higher in HRAY (mean: 4.23, CrI: 2.32–7.33) than in LRAY (mean: 1.95, CrI: 0.95–3.8), with support for a significant difference between HRAY and LRAY (posterior probability of 0.995 above the significance threshold; Figure 5a). Fox abundance was also higher in HRAY (mean: 2.62, CrI: 1.49–4.15) than in LRAY (mean: 1.45, CrI: 0.68–2.49), with support for a significant difference between HRAY and LRAY (posterior probability of 0.976 being above the significance threshold; Figure 5b). Finally, the marten abundance was not higher in HRAY (mean: 1.3, CrI: 0.56–2.54) than in LRAY (mean: 0.55, CrI: 0.09–1.30), with no support for a significant difference between HRAY and LRAY (posterior probability of 0.943 being below the significance threshold; Figure 5c).

## 4 DISCUSSION

The results support our second hypothesis that rodents influence wood warbler nest survival indirectly by triggering a numerical response of rodent‐hunting predators, which incidentally depredate wood warbler nests while hunting rodents. Rodent‐hunting predators, specifically pine marten and red fox, were the principal mammalian nest predators of wood warbler nests in Switzerland, while rodents depredated only a minute portion of nests.

That rodent abundance is an important driver of wood warbler behavior, and ecology has been shown in previous studies at several scales. Wood warbler spring settlement at the territory scale (Switzerland; Pasinelli et al., 2016), population size at the forest stand scale (Białowieża National Park; Wesołowski et al., 2009), and abundance at the landscape scale (Poland; Szymkowiak & Kuczyński, 2015; Germany; A. Grendelmeier, M. Flade & G. Pasinelli, unpublished data) were all negatively related to rodent abundance. In the present study, we additionally found that also a component of reproductive success negatively correlated with rodent abundance. Nest survival was lower in HRAY than in LRAY (Figure 4a). To our knowledge, this is the first study documenting a negative relationship between wood warbler nest survival and rodents. Analyzing short‐term data, Wesołowski et al. (2009) initially found a positive relationship between wood warbler nest loss and rodent abundance, which disappeared, however, when analyzing a longer time series of data. Evidence for a correlation between passerine nest survival and rodents has also been reported from other systems and species. For example, Schmidt and Ostfeld (2003) found that daily nest mortality rate of veeries (*Catharus fuscescens*), red‐eyed vireos (*Vireo olivaceus*), and wood thrushes (*Hylocichla mustelina*) was positively related to rodent density in the Hudson Valley, New York, USA. Our study and others (Clotfelter et al., 2007; Grendelmeier et al., 2015; Jȩdrzejewska & Jȩdrzejewski, 1998; Schmidt & Ostfeld, 2003, 2008; Szymkowiak & Kuczyński, 2015; Wesołowski et al., 2009) exemplify how strongly life history of some songbird species is affected by rodents, negatively influencing reproduction, even after rodent‐mediated partial habitat avoidance (leading to reduced settlement) has already operated.

Even though wood warbler reproduction is related to rodent abundance, we could not confirm an important direct role of rodent predation and thus found no support for our first hypothesis. Rodents have been documented to predate and/or destroy nests of several bird species in general (Kirkpatrick & Conway, 2010; Walankiewicz, 2002) and have been suspected to be an important nest predator of wood warbler nests via circumstantial indirect evidence (Mildenberger, 1940; Wesołowski, 1985; Wesołowski et al., 2009). However, direct evidence from using nest cameras in Northern Switzerland (this study, Grendelmeier et al., 2015), Marburg, Germany (Stelbrink, 2016), Wales, UK (Mallord et al., 2012), England, UK (Bellamy et al., 2018; Maziarz et al., 2017) and Białowieża National Park, Poland (Maziarz et al., 2018) now suggest that rodents are not important predators of wood warbler nests. Low predation by rodents is astonishing, considering that rodents were filmed moving around and even inspecting nests without predation occurring on many occasions (Maziarz et al., 2018). Whether wood warblers regard rodents as threat, even though they are not (compared to e.g., RHP), or use rodent abundance as a proxy for predation risk in general remains to be tested.

Our results suggest that rodents influence wood warbler reproduction indirectly by triggering a numerical, but not a behavioral response of rodent‐hunting predators. In line with predictions of hypothesis 2 about a numerical response, we found that nest survival was negatively related to rodent abundance and that the proportion of nests predated by RHP was higher in HRAY than in LRAY (Figure 4b). Furthermore, we have direct evidence for a numerical response of RHM in relation to rodent abundance (Figure 5a). Numerical responses of predators to increased rodent abundances have been revealed in various ecosystems and various predator–prey systems. In a temperate forest ecosystem in New York, USA, abundances of Cooper's (*Accipiter cooperii*) and sharp‐shinned hawks (*A. striatus*) both showed a positive relationship with rodent densities the previous year (Schmidt & Ostfeld, 2003). In high arctic Greenland, arctic fox (*Alopex lagopus*), stoat (*Mustela erminea*), long‐tailed skua (*Stercorarius longicaudus*), and snowy owl (*Nyctea scandiaca*) exhibited numerical responses in relation to densities of the collared lemming (*Dicrostonyx groenlandicus*), albeit to varying degrees (Gilg et al., 2006). And in semiarid neotropical Chile, five out of 10 studied predator species (four and one species from the orders Falconiformes and Strigiformes, respectively) showed numerical responses in relation to densities of small mammals (Jaksić, Jiménez, Castro, & Feinsinger, 1992). In Europe, common buzzards and tawny owls (Jȩdrzejewski & Jȩdrzejewska, 1993; Jȩdrzejewski, Jȩdrzejewska, et al., 1994; Jȩdrzejewski, Szymura, & Jȩdrzejewska, 1994) as well as Tengmalm's owl (*Aegolius funereus*) (Korpimäki, 1985) are also known to be strongly linked to rodent abundances.

Red fox and martens are generalist predators and use rodents as food source. A numerical response through a demographically based population increase in HRAY is not possible for any of the three species due to their long reproductive cycles. Though red foxes may copulate as early as December, juvenile independence and dispersal do not occur until early fall of the following year (Heptner & Sludskii, 2002). Stone and pine marten copulate in late summer, but due to delayed implantation, parturition does not occur until spring in the following year (Heptner & Sludskii, 2002). We thus most likely observed a movement‐based, or aggregative numerical response (DeCesare, Hebblewhite, Robinson, & Musiani, 2010; Holt & Kotler, 1987), meaning that RHM were attracted to rodent‐rich forest floors. Increased occurrence of predators hunting for rodents on the forest floor might then have resulted in increased encounter rates of incidental prey such as wood warbler nests. Similar patterns were found for Scottish moorlands, where aggregative numerical responses of buzzards to increased vole numbers were associated with higher incidental predation of grouse (Francksen et al., 2017). In the Canadian arctic tundra, trophic interactions also revolved around a shared predator, the arctic fox, and three prey species (McKinnon et al., 2013). Arctic foxes showed an aggregative numerical response to geese nests as alternative prey at low lemming abundances (main prey) and thereby increasingly predated artificial shorebird nests (incidental prey).

Lastly, we found no evidence that nest predation by jays and nRHP was related to rodent abundance, and hence, hypotheses four and five were not supported. Considering that predators evaluated in these two hypotheses are not linked to rodents, the absence of a correlation is not surprising. Nevertheless, jays are important predators of wood warbler nests, but the jay's predatory impact does not appear to differ between HRAY and LRAY. This pattern may result from the risk‐sensitive antipredator strategy suggested by Szymkowiak and Kuczyński (2015), where wood warblers avoid settling in areas with high densities of jays during years with low rodent abundance and thus minimize predation by the two most important predator groups (RHP and jays).

To conclude, mast seeding events in our study system triggered a demographically based numerical increase of rodent populations (Jensen, 1982; Pucek et al., 1993), which leads to an aggregative numerical response of RHM to rodents (primary prey) and to increased incidental predation on wood warbler nests (incidental prey). The documented inverse relationship between rodent abundance and wood warbler population size and the trophic interactions found in this study seem to fit the pattern described by apparent competition (Holt, 1977), where an increase of one species coincides with the decrease of another species mediated by shared predators (DeCesare et al., 2010). In our study system, high rodent abundances were accompanied by low wood warbler abundances in HRAY, and vice versa in LRAY, with the RHM red fox and martens as shared predators. The global wood warbler population (Vickery et al., 2014) appears to be decreasing overall, with strong declines in some countries, including Switzerland (Knaus et al., 2018). These patterns are accompanied by growing evidence that mast seeding frequency (Övergaard et al., 2007; Paar et al., 2011; Vetter, Ruf, Bieber, & Arnold, 2015) and overall seed production (Callahan et al., 2008; Gatter, 2000; Hilton & Packham, 2003) have increased over the past century. Increasingly, frequent mast years and larger seed crops providing more food to seed consumers may have led to overall higher rodent populations in the past decades and subsequently to increasing nest predation pressure by RHP showing aggregative numerical responses to forest rodents. This hypothesis stands in contrast to Europe‐wide population declines of vole species occupying nonforest habitats (Cornulier et al., 2013). However, the decline of the latter vole species is likely linked to the intensification of agriculture practices throughout Europe. To our knowledge, no comprehensive work reporting trends for forest‐inhabiting rodents in Europe has been published. If forests have provided increasingly richer hunting grounds for predators, mast‐mediated apparent competition may have increased and thus rendered formerly occupied habitats less suitable for species such as wood warblers. Furthermore, when settlement does occur, higher predator occurrence may lead to reduced fitness due to increased predation pressure (Martin, 1995) or even just perceived predation risk (Zanette, White, Allen, & Clinchy, 2011). Nomadism, a possible mechanism to minimize the effects of apparent competition with rodents by causing wood warblers to seek out regions with few rodents (Wesołowski et al., 2009), may coincidentally have decreased in effectiveness. Changes in the strength of apparent competition between rodents and wood warblers via their shared predators could thus at least in part explain the decline of wood warblers (Vickery et al., 2014). This possibility warrants more research, as apparent competition has been invoked as a proximate mechanism underlying endangerment of some species (DeCesare et al., 2010).

## ETHICAL APPROVAL

All applicable institutional and national guidelines for the care and use of animals were followed.

## CONFLICT OF INTEREST

All authors declare no conflict of interest.

## AUTHOR CONTRIBUTIONS

AG, RA, and GP conceived the study; AG collected and analyzed the data; AG and GP wrote the manuscript; RA critically commented manuscript drafts.

## DATA ACCESSIBILITY

Data are archived on Dryad Digital Repository under the https://doi.org/10.5061/dryad.860s8m3; Data files: Grendelmeier et al. (2018)—Ecol & Evol—Data.
