# Supplementary material for: Numerical response of mammalian carnivores to rodents affects bird reproduction in temperate forests: A case of apparent competition?
Source: Ecol Evol. 2018 Nov 20;8(23):11596–608. doi: 10.1002/ece3.4608 (PMC6303777; doi:10.1002/ece3.4608)
Supplement: Supplementary file 1 [file ECE3-8-11596-s001.docx]

**Supporting information**

**Table S1 Number of plot pairs in which rodents were captured**. Plot pair refers to one wood warbler territory and its paired control area without wood warbler occurrence 200 m in a random direction. Given are the study area with canton in brackets, study area coordinates and year. Finally “number of nests surveyed” refers to the number of nests that were camera-monitored and contributed to the data set for nest survival models. If for one study area certain years are not shown, neither nests surveys nor rodent captures took place. * in 2012 wood warbler numbers were very low, so we captured rodents on plots corresponding to territories and paired control areas of previous years.

| **Area** | **Coordinate** | **Year** | **Number of plot pairs captured in** | **Number of nests surveyed** |
| --- | --- | --- | --- | --- |
| Bänkerjoch (AG) | N 47° 26.2' E 8° 2.1' | 2010 | 1 | 1 |
|  |  | 2011 | 0 | 1 |
| Belchen (SO) | N 47° 21.7' E 7° 48.6' | 2011 | 3 | 4 |
| Blauen (BL) | N 47° 27.6' E 7° 31.7' | 2010 | 0 | 3 |
|  |  | 2011 | 3 | 12 |
|  |  | 2012 | 4* | 1 |
|  |  | 2013 | 0 | 5 |
|  |  | 2014 | 0 | 7 |
| Dittingen (BL) | N 47° 26.9' E7° 28.8' | 2010 | 0 | 1 |
|  |  | 2011 | 0 | 2 |
| Erschwil (SO) | N 47° 22.4 E 7° 33.3' | 2012 | 2 | 2 |
|  |  | 2013 | 0 | 8 |
|  |  | 2014 | 0 | 6 |
| Gündelhart (TG) | N 47° 38.7' E 8° 56.4' | 2010 | 1 | 1 |
| Glarus (GL) | N 47° 2.3' E 9° 5.2' | 2010 | 4 | 6 |
|  |  | 2011 | 4 | 4 |
|  |  | 2012 | 4 | 5 |
| Homberg (SO) | N 47° 21.5', E 7° 50.9' | 2011 | 4 | 5 |
|  |  | 2012 | 2 | 2 |
| Hochwald (SO) | N 47° 27.5' E 7° 39.7' | 2011 | 3 | 3 |
|  |  | 2013 | 0 | 1 |
| Kleinlützel (SO) | N 47° 26.3' E 7° 25.9' | 2010 | 4 | 5 |
|  |  | 2011 | 4 | 6 |
|  |  | 2012 | 4 | 6 |
|  |  | 2013 | 4 | 4 |
|  |  | 2014 | 5 | 8 |
|  |  | 2015 | 4 | 5 |
| Langenbruck (BL) | N 47° 21.5' E 7° 47.3' | 2010 | 3 | 5 |
|  |  | 2011 | 0 | 2 |
|  |  | 2012 | 2* | 0 |
|  |  | 2013 | 1 | 1 |
| Lauwil (BL) | N 47° 22.4' E 7° 39.7' | 2010 | 4 | 7 |
|  |  | 2011 | 5 | 12 |
|  |  | 2012 | 2 | 0 |
|  |  | 2013 | 4 | 4 |
|  |  | 2014 | 1 | 10 |
|  |  | 2015 | 5 | 12 |
| Montsevelier (JU) | N 47° 22.1’ E 7° 29.2’ | 2010 | 2 | 3 |
|  |  | 2011 | 3 | 8 |
|  |  | 2012 | 4 | 4 |
|  |  | 2013 | 6 | 6 |
|  |  | 2014 | 4 | 12 |
|  |  | 2015 | 2 | 4 |
| Oltingen (BL) | N 47° 25.8’ E 7° 56.5’ | 2010 | 1 | 1 |
| Staffelegg (AG) | N 47° 25.4’ E 8° 4.1’ | 2010 | 1 | 1 |
|  |  | 2011 | 0 | 1 |
|  |  | 2012 | 1 | 1 |
| Scheltenpass (SO) | N 47° 20.8’ E 7° 37.1’ | 2010 | 6 | 14 |
|  |  | 2011 | 2 | 4 |
|  |  | 2012 | 0 | 2 |
|  |  | 2013 | 0 | 1 |
|  |  | 2014 | 0 | 7 |
|  |  | 2015 | 6 | 9 |

**Table S2 Capture probabilities (p) and density estimates (N) of rodents calculated with program CAPTURE (v6.0) assuming a closed population with data from 2010.** M(o): null model, M(t): time model, M(h): heterogeneity model, M(th): time-heterogeneity model.

| Territory | Animals captured | Capture probability (p) | Density estimate (N) | SE | L 95% CI | U 95% CI | Most appropriate model |
| --- | --- | --- | --- | --- | --- | --- | --- |
| BJ01 | 30 | 0.242 | 37 | 3.9 | 33 | 49 | M(o) |
| BJ11 | 36 | 0.145 | 58 | 10.4 | 46 | 89 | M(o) |
| GH01 | 8 | 0.055 | 27 | 22.5 | 12 | 126 | M(o) |
| GH11 | 35 | 0.1 | 85 | 15 | 64 | 123 | M(h) |
| GL01 | 23 | 0.192 | 34 | 6.4 | 27 | 54 | M(t) |
| GL02 | 4 | 0.33 | 4 | 2.5 | 4 | 4 | M(h) |
| GL03 | 4 | 0.167 | 6 | 1.8 | 5 | 13 | M(h) |
| GL04 | 24 | 0.295 | 26 | 1.5 | 25 | 31 | M(t) |
| GL11 | 18 | 0.22 | 21 | 2.3 | 19 | 29 | M(t) |
| GL12 | 17 | 0.307 | 19 | 2.6 | 18 | 31 | M(th) |
| GL13 | 12 | 0.119 | 21 | 5.7 | 16 | 39 | M(h) |
| GL14 | 17 | 0.199 | 23 | 4.2 | 18 | 36 | M(o) |
| KL02 | 16 | 0.116 | 33 | 8.1 | 23 | 57 | M(h) |
| KL03 | 17 | 0.138 | 27 | 6.9 | 20 | 50 | M(t) |
| KL04 | 8 | 0.25 | 10 | 3.2 | 9 | 25 | M(th) |
| KL05 | 16 | 0.193 | 22 | 4.3 | 17 | 36 | M(o) |
| KL12 | 20 | 0.122 | 37 | 7.9 | 27 | 60 | M(o) |
| KL13 | 22 | 0.148 | 33 | 6.8 | 26 | 55 | M(t) |
| KL14 | 18 | 0.13 | 29 | 7.8 | 22 | 56 | M(t) |
| KL15 | 23 | 0.067 | 65 | 13.3 | 47 | 100 | M(h) |
| LB01 | 7 | 0.115 | 13 | 7.6 | 8 | 47 | M(th) |
| LB04 | 9 | 0.25 | 10 | 2.6 | 10 | 25 | M(h) |
| LB05 | 5 | 0.25 | 6 | 1.8 | 6 | 15 | M(h) |
| LB11 | 3 | 0.333 | 3 | 0.6 | 3 | 3 | M(o) |
| LB14 | 7 | 0.128 | 13 | 4.3 | 9 | 28 | M(h) |
| LB15 | 19 | 0.2 | 23 | 2.9 | 20 | 33 | M(t) |
| LW01 | 9 | 0.233 | 10 | 2.6 | 10 | 25 | M(h) |
| LW03 | 8 | 0.233 | 10 | 2.6 | 9 | 22 | M(h) |
| LW06 | 6 | 0.25 | 6 | 2.2 | 6 | 19 | M(h) |
| LW07 | 5 | 0.233 | 5 | 2.2 | 5 | 20 | M(h) |
| LW11 | 7 | 0.262 | 7 | 0 | 7 | 7 | M(t) |
| LW13 | 11 | 0.278 | 11 | 0.7 | 11 | 14 | M(t) |
| LW16 | 20 | 0.085 | 45 | 18 | 28 | 108 | M(t) |
| LW17 | 13 | 0.308 | 13 | 0 | 13 | 13 | M(t) |
| MS01 | 10 | 0.235 | 12 | 3.5 | 11 | 29 | M(th) |
| MS02 | 9 | 0.208 | 12 | 2.3 | 10 | 20 | M(h) |
| MS11 | 25 | 0.235 | 30 | 3.1 | 27 | 40 | M(t) |
| MS12 | 38 | 0.112 | 71 | 15.6 | 52 | 117 | M(t) |
| OL01 | 19 | 0.173 | 25 | 4.5 | 21 | 41 | M(t) |
| OL11 | 34 | 0.059 | 104 | 16.7 | 79 | 145 | M(h) |
| SE01 | 23 | 0.185 | 31 | 4.8 | 26 | 46 | M(t) |
| SE11 | 22 | 0.173 | 31 | 5.5 | 25 | 48 | M(t) |
| SP02 | 6 | 0.263 | 7 | 2.1 | 7 | 19 | M(th) |
| SP07 | 2 | 0.25 | 2 | 0.9 | 2 | 2 | M(o) |
| SP08 | 3 | 0.133 | 5 | 1.8 | 4 | 12 | M(h) |
| SP09 | 6 | 0.167 | 10 | 5.7 | 7 | 37 | M(th) |
| SP11 | 15 | 0.22 | 17 | 1.8 | 15 | 25 | M(t) |
| SP13 | 8 | 0.292 | 10 | 3 | 9 | 25 | M(th) |
| SP22 | 6 | 0.097 | 12 | 4.2 | 8 | 26 | M(h) |
| SP27 | 4 | 0.167 | 6 | 1.8 | 5 | 13 | M(h) |
| SP28 | 4 | 0.167 | 7 | 5 | 5 | 32 | M(th) |
| SP29 | 15 | 0.123 | 27 | 10.6 | 18 | 67 | M(th) |
| SP31 | 6 | 0.271 | 8 | 1.8 | 7 | 15 | M(h) |
| SP33 | 21 | 0.177 | 31 | 5.8 | 25 | 49 | M(h) |
